# The Antioxidant and Neuroregenerative Effects of Thymoquinone in a Rat Intracerebral Hemorrhage Model

**DOI:** 10.3390/biomedicines14051009

**Published:** 2026-04-29

**Authors:** Khamim Thohari, Asra Al Fauzi, Djoko Agus Purwanto

**Affiliations:** 1 Doctoral Program of Medical Science, Universitas Airlangga, Surabaya 60131, Indonesia; khamim.thohari-2023@fk.unair.ac.id; 2 School of Medicine, Universitas Ciputra, Surabaya 60219, Indonesia; 3 Department of Neurosurgery, Faculty of Medicine, Universitas Airlangga, Universitas Airlangga Hospital, Surabaya 60131, Indonesia; 4 Department of Pharmaceutical Chemistry, Faculty of Pharmacy, Universitas Airlangga, Surabaya 60115, Indonesia; djokoagus@ff.unair.ac.id

**Keywords:** thymoquinone, intracerebral hemorrhage, oxidative stress, NRF2, SOD, MDA, neuronal survival, neuroprotection

## Abstract

**Background/Objectives:** Intracerebral hemorrhage (ICH) is a severe subtype of stroke characterized by extensive secondary brain injury driven by oxidative stress, inflammation, and progressive neuronal loss, leading to poor neurological outcomes. Thymoquinone, a bioactive compound derived from Nigella sativa, has demonstrated potent antioxidant and neuroprotective properties, but its integrated effects in hemorrhagic stroke remain insufficiently explored. This study aimed to evaluate the antioxidant and neuroregenerative effects of thymoquinone in a rat model of ICH. **Methods:** Male Wistar rats with experimentally induced ICH were randomized into untreated controls and two treatment groups receiving thymoquinone (150 mg/kg and 250 mg/kg) for three consecutive days. Oxidative injury and antioxidant responses were assessed using membrane blebbing, malondialdehyde (MDA), superoxide dismutase (SOD) activity, and nuclear factor erythroid 2-related factor 2 (NRF2) expression, while neuroprotection was evaluated by neuronal counts in perihematomal tissue. **Results:** Thymoquinone treatment significantly reduced membrane blebbing and MDA levels, while markedly increasing SOD activity and NRF2 expression in a dose-dependent manner. These biochemical improvements were accompanied by significant preservation of neuronal morphology and increased neuronal survival, with the 250 mg/kg dose showing the strongest effects. **Conclusions:** In conclusion, thymoquinone confers robust antioxidant and neuroprotective benefits in experimental ICH and represents a promising candidate for mitigating secondary brain injury following intracerebral hemorrhage.

## 1. Introduction

Intracerebral hemorrhage is considered one of the most serious types of strokes, with a significant impact on global neurological morbidity and mortality [1]. Although intra-cerebral hemorrhage accounts for a smaller proportion of stroke cases, it often results in more severe clinical outcomes. This is largely due to the immediate mechanical damage caused by the hematoma, which is subsequently followed by a complex cascade of secondary injury processes. Emerging evidence suggests that these secondary mechanisms—including oxidative stress, inflammatory responses, excitotoxicity, mitochondrial dysfunction, and progressive neuronal loss—play a crucial role in determining long term neurological deficits [2].

Oxidative stress plays a central role in secondary brain injury following intracerebral hemorrhage. The breakdown of extravasated blood releases hemoglobin and iron, which promote excessive production of reactive oxygen species (ROS), leading to lipid peroxidation, mitochondrial dysfunction, and neuronal membrane damage [3]. These processes are reflected by increased malondialdehyde levels, reduced activity of endogenous antioxidant enzymes such as superoxide dismutase, and structural changes including membrane blebbing. Impaired activation of nuclear factor erythroid 2-related factor 2 (NRF2) further compromises antioxidant defenses, contributing to progressive neuronal loss. Therefore, assessment of these parameters provides a comprehensive evaluation of oxidative imbalance after intracerebral hemorrhage [4]. Given the central role of NRF2, this pathway represents a potential therapeutic target, and compounds such as thymoquinone may exert neuroprotective effects by enhancing endogenous antioxidant responses.

Besides oxidative stress, neuroinflammation represents another major factor driving neurological deterioration after intracerebral hemorrhage. Blood breakdown products and damaged cellular components activate microglia, leading to the release of proinflammatory mediators such as TNF-α, IL-1β, and components of the NLRP3 inflammasome [5]. In parallel, matrix metalloproteinases, particularly MMP-9, are upregulated and contribute to blood–brain barrier disruption, increased vascular permeability, and exacerbation of neuronal injury [6]. The resulting inflammatory cascade further enhances neuronal vulnerability and aggravates tissue damage. Therefore, therapeutic agents that can simultaneously attenuate oxidative stress and suppress inflammatory pathways hold significant potential for modifying the progression of secondary injury.

Thymoquinone, the main bioactive compound found in Nigella sativa, has attracted considerable attention because of its diverse pharmacological effects. Recent studies have shown that thymoquinone exhibits strong antioxidant activity, including the ability to reduce lipid peroxidation, enhance the activity of endogenous antioxidant enzymes, maintain cellular membrane stability, and activate protective signaling pathways such as nuclear factor erythroid 2-related factor 2 (NRF2) [7]. Thymoquinone has also been reported to exhibit strong anti-inflammatory properties. This compound can reduce the expression of proinflammatory mediators such as tumor necrosis factor-α and interleukin-1β, inhibit the activation of the NLRP3 inflammasome, and limit matrix metalloproteinase-9-related tissue degradation [8]. These collective properties indicate that thymoquinone may effectively target multiple aspects of secondary brain injury following intracerebral hemorrhage.

Beyond its antioxidant and anti-inflammatory capacities, recent studies suggest that thymoquinone may also enhance neuronal recovery. Evidence indicates that thymoquinone can support neuronal survival, promote neurogenesis, regulate apoptotic pathways, and stimulate the expression of molecular regulators involved in cellular repair and differentiation [9]. Considering that neuronal regeneration is limited after intracerebral hemorrhage, interventions with dual protective and regenerative functions are particularly valuable.

Despite these promising findings, comprehensive investigations examining the integrated antioxidant, anti-inflammatory, and neuroregenerative effects of thymoquinone specifically in an intracerebral hemorrhage model remain limited. Most available research focuses on ischemic injury or generalized neurotoxicity, leaving important questions regarding the role of thymoquinone in the unique biochemical environment of hemorrhagic stroke. Given that oxidative stress, inflammation, and neuronal loss occur concurrently and interact synergistically after intracerebral hemorrhage, there is a need for experimental studies that simultaneously evaluate these parameters.

Therefore, the present study aims to investigate the antioxidant and neuroregenerative effects of thymoquinone in an intracerebral hemorrhage rat model by assessing key indicators of oxidative stress, membrane injury, endogenous antioxidant response, and neuronal survival. A deeper understanding of these mechanisms may contribute to the development of thymoquinone as a potential therapeutic agent capable of mitigating secondary injury and enhancing neural recovery following intracerebral hemorrhage.

## 2. Materials and Methods

### 2.1. Experimental Design and Animals

This experimental laboratory study employed a controlled post-test-only design to evaluate the antioxidant and neuroregenerative effects of thymoquinone (TQ) in a rat model of intracerebral hemorrhage (ICH). Adult male Wistar rats (*Rattus norvegicus*) weighing 200–250 g and aged 8–10 weeks were obtained from the Animal Research Facility, Faculty of Medicine, Universitas Airlangga, and maintained under standard laboratory conditions (22–25 °C, 50–60% humidity, 12 h light/dark cycle) with ad libitum access to food and water following a 7-day acclimatization period. Animal health and environmental conditions were monitored daily to ensure experimental stability and to minimize stress-related confounders.

The sample size was determined using Federer’s formula for experimental studies to ensure an adequate number of subjects per group for statistical analysis. Based on this approach, a minimum of 10 animals per group was required; therefore, 11 rats were included in each group to increase statistical robustness and account for potential variability. A formal power analysis was not performed; however, the sample size is consistent with previous experimental studies using similar intracerebral hemorrhage models [10,11]. Rats that exhibited signs of illness prior to the induction of intracerebral hemorrhage through homologous blood intraparenchymal injection were excluded from the study.

### 2.2. Induction of Intracerebral Hemorrhage

The ICH model was induced using a stereotaxic-guided intracerebral autologous blood injection technique under anesthesia. Animals were anesthetized by intraperitoneal injection of a ketamine–xylazine mixture (ketamine 100 mg/kg and xylazine 10 mg/kg) under strict aseptic conditions. This method is well established and widely used in experimental ICH studies [12,13]. Briefly, a burr hole was drilled at predetermined coordinates relative to bregma, and autologous whole blood was slowly injected into the striatal region to produce focal hemorrhage. The injection needle was retained in situ for several minutes to prevent reflux, after which the incision was sutured and animals were allowed to recover in warmed cages under close postoperative observation. To ensure consistency of the intracerebral hemorrhage model, all procedures were performed using the same stereotaxic coordinates and injection volume under standardized conditions. Successful induction of intracerebral hemorrhage was confirmed by histopathological identification of hematoma formation in the perihematomal region. No animals were excluded due to failure of model induction.

### 2.3. Experimental Groups and Treatment

Following hemorrhage induction, animals were randomly allocated into three experimental groups, namely untreated ICH controls, ICH treated with TQ 150 mg/kg, and ICH treated with TQ 250 mg/kg, The use of a high dose of thymoquinone (250 mg/kg) in this study was supported by previous experimental findings showing that oral administration of thymoquinone at doses up to 250 mg/kg/day in rats is well tolerated and associated with significant anti-inflammatory and antioxidant effects [14]. This supports the rationale for evaluating its maximal therapeutic potential in experimental brain injury. Thymoquinone (Sigma-Aldrich, St. Louis, MO, USA) was freshly prepared prior to administration and dissolved in corn oil as the vehicle. It was administered once daily by oral gavage for three consecutive days starting 2 h after intracerebral hemorrhage (ICH) induction. Control animals that did not receive thymoquinone were administered an equivalent volume of corn oil following the same schedule. Treatment was initiated 2 h after ICH induction to target the early phase of secondary brain injury, during which oxidative stress and inflammatory responses are rapidly activated. Previous experimental studies have demonstrated that reactive oxygen species production, lipid peroxidation, and microglial activation begin within the first few hours following hemorrhage. Therefore, early intervention at this stage is critical to attenuate the cascade of secondary injury and prevent progression of neuronal damage. Consistent with experimental brain injury models, neuroprotective agents including thymoquinone have been shown to be most effective when administered within the early post-injury window [15].

### 2.4. Tissue Collection and Processing

At the end of the intervention period, animals were deeply anesthetized. For histopathological and immunohistochemical analysis, animals were transcardially perfused with phosphate-buffered saline followed by 4% paraformaldehyde to preserve tissue architecture. The brains were then harvested, post-fixed, processed for paraffin embedding, and serially sectioned at the perihematomal region. Sections were subjected to routine histopathological staining and immunohistochemical procedures using standardized protocols to ensure consistency across samples and minimize technical variability. All animals in each group (n = 11) were included in both analyses.

### 2.5. Histopathological Assessment

Oxidative membrane injury was evaluated histologically through the assessment of membrane blebbing as an indicator of lipid membrane destabilization. Membrane blebbing was defined as the presence of spherical protrusions of the plasma membrane associated with cytoskeletal detachment. Histological examination was performed on hematoxylin and eosin (H&E)-stained brain sections and evaluated visually using light microscopy [16]. Blebbing was identified based on morphological criteria and quantified by counting neurons exhibiting membrane protrusions in multiple randomly selected high-power fields within the perihematomal region.

However, it is important to note that membrane blebbing is not specific to oxidative stress and may also occur in conditions such as apoptosis, mechanical stress, and cytoskeletal disruption. Therefore, in this study, membrane blebbing was interpreted in conjunction with biochemical markers of oxidative stress (MDA and SOD) and NRF2 signaling to provide a more comprehensive assessment of oxidative injury and neuronal damage.

To further evaluate the impact of oxidative injury on neural tissue, neuronal survival and tissue integrity were assessed by quantitative neuron counts in perihematomal areas using hematoxylin and eosin staining and established morphological criteria for intact neurons. For each animal, neurons were counted in multiple randomly selected high-power fields by two independent observers who were blinded to the treatment allocation. This approach provided an estimate of neuronal preservation based on morphological characteristics; however, it does not distinguish between viable, functionally impaired, or dying neurons, nor does it differentiate between mechanisms of cell death such as apoptosis and necrosis.

### 2.6. Immunohistochemistry

Immunohistochemical staining was performed to evaluate the expression and localization of oxidative stress-related markers. MDA expression was assessed using an anti-malondialdehyde antibody (Antibodies.com, Cambridge, UK; Cat. No. A82233), and SOD1 expression was evaluated using an anti-SOD1 antibody (Santa Cruz Biotechnology, Dallas, TX, USA; Cat. No. sc-1011523). NRF2 expression was assessed using an anti-NRF2 antibody (Santa Cruz Biotechnology, Dallas, TX, USA; Cat. No. sc-28379). Semi-quantitative analysis was conducted based on staining intensity and the proportion of positive cells to estimate protein expression levels in tissue.

### 2.7. Statistical Analysis

Animals were allocated for both Immunohistochemistry and histological analyses, with equal representation across groups. All histological and immunohistochemical assessments were performed under blinded conditions to reduce measurement bias. Data were expressed as mean ± standard deviation. Normality of data distribution was assessed using the Shapiro–Wilk test, and homogeneity of variances was evaluated using Levene’s test. Group differences were analyzed using one-way analysis of variance for normally distributed data, whereas non-parametric comparisons were conducted using the Mann–Whitney U test when assumptions for parametric testing were not met. Appropriate post hoc analyses were applied to identify intergroup differences and to determine the most effective dose of thymoquinone, with statistical significance set at *p* < 0.05.

## 3. Results

Assumption testing demonstrated that all oxidative stress and neuronal parameters fulfilled the statistical requirements for parametric analysis. Homogeneity testing using Levene’s test indicated that membrane blebbing, MDA, SOD, NRF2, and neuronal counts each produced *p*-values greater than 0.05, confirming uniform variance across the three groups. This consistency suggests that variability in oxidative and neuronal outcomes was not influenced by unequal group dispersion.

Similarly, the Shapiro–Wilk test showed that all parameters followed a normal distribution, as reflected by *p*-values exceeding the 0.05 significance threshold. The normality of membrane injury indicators (membrane blebbing), oxidative stress markers (MDA), antioxidant responses (SOD and NRF2), and neuronal survival data indicates that the sample distribution met the assumptions of Gaussian behavior required for parametric evaluation.

Collectively, the results of the homogeneity and normality tests confirm that oxidative stress and neuronal parameters were statistically appropriate for subsequent analysis using One-Way ANOVA, ensuring reliability and validity in interpreting the effects of Thymoquinone on antioxidant defenses and neuronal preservation in the ICH model.

As shown in Table 1, thymoquinone administration was associated with significant improvements in oxidative stress markers and neuronal survival in the ICH model. Membrane blebbing was markedly reduced in a dose-dependent manner, with the lowest values observed in the TQ 250 mg/kg group compared to the control group (3.55 ± 1.57 vs. 6.70 ± 1.64; *p* < 0.001). Similarly, the number of MDA-positive cells was significantly decreased following thymoquinone treatment, indicating attenuation of lipid peroxidation, with the greatest reduction observed at 250 mg/kg (4.55 ± 1.64 vs. 10.40 ± 1.58 in controls; *p* < 0.001).

In contrast, antioxidant defense parameters showed a significant increase. The number of SOD-positive cells was elevated in both treatment groups, with the highest value observed in the TQ 250 mg/kg group (11.27 ± 1.74 vs. 5.50 ± 1.27 in controls; *p* < 0.001). A similar trend was observed for NRF2-positive cells, which increased substantially following thymoquinone administration (11.91 ± 1.30 vs. 4.10 ± 1.45 in controls; *p* < 0.001), suggesting activation of endogenous antioxidant pathways.

Neuronal survival was also significantly improved, as reflected by increased neuron counts in the treatment groups, particularly at the higher dose (11.36 ± 2.11 vs. 4.20 ± 2.10 in controls; *p* < 0.001). Post hoc analysis confirmed that the 250 mg/kg dose produced significantly greater effects compared to 150 mg/kg across all parameters (*p* < 0.05). Overall, these findings demonstrate a consistent dose-dependent neuroprotective and antioxidant effect of thymoquinone in the ICH model.

**Figure 1:**
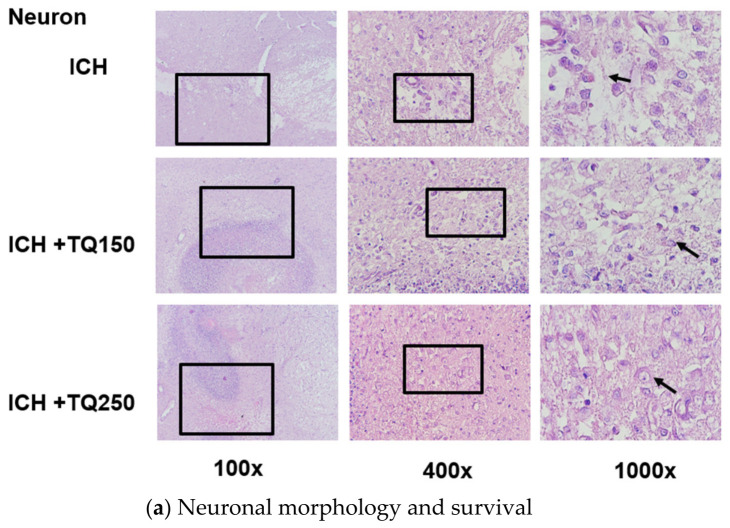

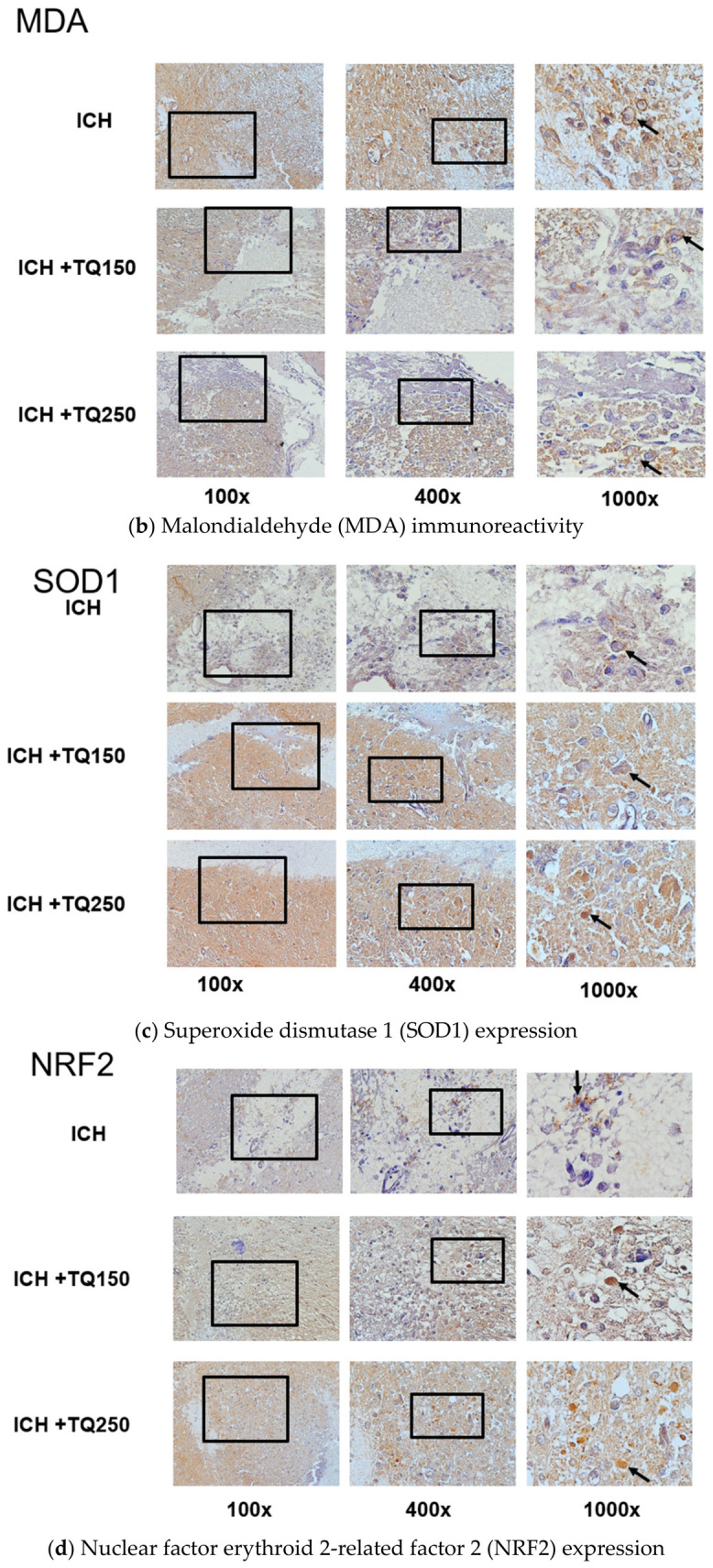
Representative histopathological and immunohistochemical micrographs of perihematomal brain tissue in the intracerebral hemorrhage (ICH) rat model. Rows represent experimental groups (top to bottom): ICH, ICH + TQ150, and ICH + TQ250. Columns represent magnification levels (100×; 400×; 1000×). The ICH group shows intense MDA staining with reduced SOD1 and NRF2 expression and marked neuronal degeneration. Thymoquinone treatment enhances SOD1 and NRF2 immunopositivity and reduces MDA staining in a dose-dependent manner, with the 250 mg/kg group showing the strongest protective effect. Black arrows indicate degenerated neurons or positive immunoreactivity. Scale bar = 50 μm.

As illustrated in Figure 1, immunohistochemical and histopathological analyses consistently demonstrate the modulatory effects of thymoquinone on oxidative stress and neuronal integrity in the perihematomal region. In the ICH group (Figure 1a–d, top row), marked pathological changes are evident across all magnifications, including reduced neuronal density, cellular shrinkage, and disorganized tissue architecture. These morphological alterations are accompanied by intense MDA immunoreactivity (Figure 1b), indicating severe lipid peroxidation, along with weak and diffuse SOD1 (Figure 1c) and NRF2 (Figure 1d) staining, reflecting impaired endogenous antioxidant defense.

Following thymoquinone administration, progressive improvements are observed in both oxidative stress markers and neuronal morphology. In the ICH + TQ150 group (middle row), MDA staining is reduced compared to the ICH group, while SOD1 and NRF2 immunopositivity become more prominent, as indicated by increased brown staining intensity and distribution across 100×, 400×, and 1000× magnifications. Correspondingly, neuronal structure appears partially preserved, with reduced cellular shrinkage and improved tissue organization.

These effects are more pronounced in the ICH + TQ250 group (bottom row), where MDA immunoreactivity is markedly diminished, indicating effective suppression of lipid peroxidation. In contrast, SOD1 and NRF2 expression are substantially increased, as evidenced by stronger and more widespread immunostaining. This enhancement is supported by semi-quantitative evaluation of staining intensity and the proportion of positive cells. Morphologically, neuronal preservation is most evident at this dose, characterized by larger soma, clearer nucleoplasm, and reduced signs of degeneration across all magnifications (Figure 1a).

## 4. Discussion

The findings of this study demonstrate that thymoquinone exerts significant antioxidant and neuroprotective effects in the intracerebral hemorrhage model. The validity of the statistical analysis was confirmed through assumption testing, which showed that all parameters exhibited normal distribution and homogeneous variance. These results strengthen the reliability of using ANOVA for evaluating treatment effects, consistent with recent biomedical experimental research emphasizing the importance of verifying normality and homogeneity to ensure accurate interpretation of pharmacological interventions in animal models.

Pathophysiologically, ICH induces secondary injury mechanisms—including oxidative stress, mitochondrial dysfunction, excitotoxicity, and activation of innate immune cells—which collectively increase reactive oxygen species (ROS) production [17]. The accumulation of ROS accelerates lipid peroxidation of membrane phospholipids, leading to plasma membrane instability and cytoskeletal alterations that morphologically manifest as membrane blebbing [18]. Membrane blebbing is the formation of spherical plasma membrane protrusions due to detachment from the actin cytoskeleton. It is associated with cytoskeletal disruption and is recognized as an early morphological feature of apoptosis, indicating cellular structural damage [19]. Therefore, the reduction in membrane blebbing observed in the treatment groups reflects attenuation of the secondary injury cascade that compromises membrane structural integrity.

In line with these mechanisms, our findings confirm that thymoquinone significantly attenuates cellular damage. This finding aligns with studies demonstrating that thymoquinone suppresses lipid peroxidation and stabilizes cellular membranes under conditions of heightened oxidative stress in neurological disorders [9]. The reduction in MDA levels reinforces this conclusion. Malondialdehyde (MDA), a byproduct of lipid peroxidation, is widely used as a biomarker of oxidative damage and is commonly used as an indicator of lipid peroxidation and reflects the extent of oxidative damage in brain tissue after intracerebral hemorrhage [4]. The decrease in MDA among treated animals suggests that thymoquinone effectively inhibits free radical reactions that target membrane phospholipids.

Consistently, reduced malondialdehyde (MDA) levels indicate inhibition of lipid peroxidation. MDA, a terminal product of polyunsaturated fatty acid peroxidation, is a sensitive marker of oxidative membrane damage. In ICH, hemoglobin breakdown into hemin and free iron promotes Fenton reactions, generating hydroxyl radicals that accelerate lipid peroxidation in perihematomal regions. Elevated MDA levels have been reported in ICH patients and are associated with increased bleeding volume [20]. Accordingly, the reduction in MDA in the treated groups indicates that thymoquinone effectively suppresses free radical reactions targeting membrane phospholipids.

A substantial increase in SOD activity reflects an enhancement of endogenous antioxidant capacity. Elevated SOD activity indicates an improved ability of neural tissue to neutralize superoxide radicals, which reduces the accumulation of reactive oxygen species known to induce neuronal toxicity. This observation is consistent with research showing that Thymoquinone has been reported to enhance antioxidant defenses by activating the NRF2 signaling pathway, which subsequently increases the expression of key antioxidant enzymes such as superoxide dismutase, catalase, and glutathione peroxidase [15].

After intracerebral hemorrhage, the degradation of hemoglobin releases heme and free iron, which promote excessive production of reactive oxygen species and contribute to mitochondrial dysfunction, neuronal DNA damage, and activation of apoptotic pathways [18]. Therefore, increased SOD activity reflects a crucial adaptive protective response that suppresses oxidative burden in hemorrhage-injured brain tissue. A study by Yan Zhang et al. explains that reduced SOD and catalase activities correlate with increased brain edema and neurological deficits of ICH [18], reinforcing the interpretation that increased SOD observed in this study contributes to limiting secondary brain injury.

At the molecular level, increased SOD activity may be attributed to activation of antioxidant signaling pathways, particularly PI3K/Akt and MAPK, which regulate antioxidant gene expression. Activation of PI3K/Akt promotes NRF2 nuclear translocation, leading to upregulation of SOD1, SOD2, and other antioxidant enzymes [21,22]. This mechanism supports the role of pro-survival pathways in maintaining intracellular redox homeostasis.

In the context of neuroprotection, SOD activity is closely linked to neuronal viability, with mitochondrial SOD2 playing a key role in resistance to oxidative stress. SOD2 overexpression in hemorrhagic brain injury models reduces mitochondrial membrane depolarization, cytochrome c release, and caspase-3 activation, thereby limiting neuronal apoptosis [18,23]. This supports the interpretation that increased SOD in this study contributes to mitochondrial protection and prevention of programmed cell death in perihematomal tissue.

These antioxidant effects are closely linked to NRF2 pathway activation. Thymoquinone exerts antioxidant and neuroprotective effects partly via activation of the NRF2/HO-1 pathway, enhancing endogenous antioxidant enzymes and reducing oxidative stress in neuronal cells [24]. Activation of the NRF2 pathway enhances antioxidant defenses by upregulating enzymes such as superoxide dismutase, heme oxygenase-1, and NAD(P)H quinone dehydrogenase 1, leading to reduced reactive oxygen species and lipid peroxidation. By limiting oxidative damage to membrane lipids and cytoskeletal components, NRF2 activation helps preserve membrane integrity and may reduce membrane blebbing [25]. Consequently, thymoquinone-induced NRF2 activation may contribute to reduced membrane blebbing by limiting oxidative membrane damage and stabilizing cellular structure. However, membrane blebbing is not specific to oxidative injury and may also result from apoptosis and cytoskeletal disruption.

The significant increase in NRF2 expression supports activation of cellular defense pathways by thymoquinone. NRF2 promotes transcription of antioxidant enzymes such as SOD, HO-1, and NQO1, which mitigate oxidative stress following hemorrhage [26,27]. The consistent elevation of NRF2 expression, including immunohistochemical findings, indicates enhanced antioxidant defense.

Emerging evidence indicates that NRF2 activation enhances mitochondrial turnover via the PINK1/Parkin-mediated mitophagy pathway, thereby limiting the accumulation of dysfunctional mitochondria that serve as major sources of ROS after hemorrhagic injury. Lei wang et al. reported that pharmacological activation of NRF2 promoted mitophagy, reduced mitochondrial ROS generation, and improved neuronal survival [28]. These findings provide a plausible mechanistic explanation for the coordinated increase in NRF2 expression observed in this study, suggesting that thymoquinone-mediated NRF2 activation may contribute not only to antioxidant enzyme induction but also to restoration of mitochondrial homeostasis.

These antioxidant improvements correspond to the notable rise in neuronal counts in the treatment groups. An increase in neuronal number indicates that thymoquinone effectively suppresses neurodegenerative processes and preserves neuronal viability [9,29]. This observation aligns with reports demonstrating that thymoquinone inhibits neuronal apoptosis by modulating caspase activity and reducing secondary inflammatory responses [30].

From a pathobiological standpoint, neuronal loss after ICH is primarily driven by oxidative stress, mitochondrial dysfunction, excitotoxicity, and neuroinflammation, which converge on apoptotic and necroptotic cell death pathways [1,2]. The observed increase in neuronal number therefore suggests that thymoquinone interrupts these convergent injury cascades at multiple nodes, thereby promoting neuronal survival within perihematomal regions.

From a translational perspective, the preservation of neuronal populations is closely associated with improved neurological recovery after intracerebral hemorrhage, as neuroprotective and regenerative mechanisms contribute to functional restoration in experimental models of brain injury [31]. Therefore, the robust increase in neuronal numbers observed in the thymoquinone-treated groups suggests potential functional relevance beyond biochemical improvements, supporting the candidacy of thymoquinone as an adjuvant neuroprotective agent.

Immunohistochemical analysis further validates the quantitative findings. Strong and diffuse MDA staining in the ICH group confirmed extensive lipid peroxidation, whereas a clear reduction in staining intensity was observed in the treated groups, particularly at the 250 mg/kg dose. This reduction was evident across magnifications ranging from 100× to 1000×, indicating consistent suppression of oxidative membrane damage. The enhanced SOD1 and NRF2 staining observed in the treatment groups reinforces the conclusion that thymoquinone substantially upregulates intrinsic antioxidant defenses.

Morphological evaluation demonstrated a protective effect on neuronal tissue. The ICH group showed features of neurodegeneration, including soma shrinkage, nuclear fragmentation, and tissue disorganization. In contrast, treatment groups (particularly at 250 mg/kg) exhibited more preserved neuronal morphology, with larger somata, clearer nucleoplasm, and fewer degenerative changes. These findings are consistent with evidence that thymoquinone enhances neuronal survival by reducing oxidative stress and suppressing inflammatory signaling pathways [7,8,15].

Secondary brain injury after intracerebral hemorrhage is driven by oxidative stress–mediated damage in the perihematomal region. Interventions that reduce oxidative stress have been shown to attenuate histopathological damage and improve neuronal preservation in experimental models [32]. This region is particularly vulnerable due to hemoglobin degradation and iron accumulation, which promote reactive oxygen species generation, blood–brain barrier disruption, and edema [33]. In this context, the improved neuronal morphology observed in this study suggests that thymoquinone may stabilize the perihematomal microenvironment and limit structural tissue damage.

Moreover, structural preservation of neurons may also be associated with improved mitochondrial homeostasis and reduced activation of intrinsic apoptotic pathways. Mitochondrial dysfunction plays a critical role in secondary neuronal injury following intracerebral hemorrhage, as excessive reactive oxygen species impair mitochondrial function and activate apoptotic pathways involving cytochrome c release [18,23]. The morphological integrity observed in thymoquinone-treated groups may therefore represent the histological correlate of improved mitochondrial resilience and reduced activation of apoptotic machinery.

Collectively, these findings support the interpretation that the morphological preservation of neurons in thymoquinone-treated animals reflects not only direct cytoprotection but also the broader modulation of the post-hemorrhagic microenvironment, including reduction of oxidative stress and suppression of neuroinflammatory signaling, has been associated with improved neuronal survival after intracerebral hemorrhage [34]. This integrative mode of action provides a mechanistic basis for the superior neuroprotective profile observed at higher thymoquinone doses and further substantiates its therapeutic relevance in experimental intracerebral hemorrhage.

The findings strongly suggest that thymoquinone exerts neuroprotective effects through multifactorial mechanisms, including suppression of oxidative stress, enhancement of antioxidant enzyme activity, activation of the NRF2 signaling pathway, and preservation of neuronal structure. These effects have been consistently demonstrated in recent experimental studies, where thymoquinone was shown to upregulate NRF2 expression, increase endogenous antioxidant enzymes, reduce lipid peroxidation, and improve neuronal integrity [15,35,36]. Accordingly, thymoquinone emerges as a promising therapeutic candidate for mitigating brain injury following intracerebral hemorrhage. This conclusion aligns with growing research emphasizing the importance of antioxidant-based interventions to reduce morbidity and mortality in ICH.

This study has several limitations. The duration of observation was limited to three days, which may not fully capture the long-term neuroprotective effects of thymoquinone following intracerebral hemorrhage. In addition, functional neurological outcomes were not assessed, limiting the ability to correlate biochemical and histological findings with clinical relevance. The study also did not include direct measurement of key inflammatory biomarkers such as NLRP3, TNF-α, and IL-1β, which are known to play important roles in secondary brain injury. Furthermore, the use of an experimental animal model may limit the direct translation of these findings to human clinical conditions. Finally, only two doses of thymoquinone were evaluated, and further studies are needed to determine the optimal therapeutic dose and to explore the underlying molecular mechanisms in greater detail.

## 5. Conclusions

This study demonstrates that thymoquinone provides significant neuroprotective effects in an intracerebral hemorrhage (ICH) model through robust modulation of oxidative stress markers and neuronal survival. Increasing doses of thymoquinone (150 and 250 mg/kg) consistently reduced membrane blebbing and lipid peroxidation (MDA), while markedly enhancing antioxidant defenses (SOD), NRF2 activation, and neuronal preservation. The higher dose (250 mg/kg) produced the strongest protective effect across all measured parameters, indicating a dose-dependent therapeutic response. These findings support thymoquinone as a promising antioxidant-based intervention capable of mitigating secondary brain injury following ICH.

Further investigations are recommended to validate the therapeutic potential of thymoquinone in clinically relevant ICH settings, including long-term functional outcomes, molecular pathway analysis, and dose-optimization studies. Future work should also consider integrating pharmacokinetic profiling, evaluating safety margins at higher doses, and exploring combination therapies with established neuroprotective agents. Translational studies, particularly those involving larger animal models or human cell-based systems, are needed to bridge the gap between preclinical efficacy and potential clinical application in ICH management.

## Figures and Tables

**Table 1:** Effects of Thymoquinone on Oxidative Stress and Neuronal Survival in an ICH Model.

Parameter	Control (ICH) Mean ± SD	TQ 150 mg/kg Mean ± SD	TQ 250 mg/kg Mean ± SD	ANOVA *p*-Value	Post Hoc (250 vs. 150) *p*-Value
Membrane Blebbing	6.70 ± 1.64	5.73 ± 1.49	3.55 ± 1.57	<0.001	0.003
MDA	10.40 ± 1.58	7.82 ± 2.36	4.55 ± 1.64	<0.001	0.000
SOD	5.50 ± 1.27	8.64 ± 1.63	11.27 ± 1.74	<0.001	0.000
NRF2	4.10 ± 1.45	8.91 ± 1.97	11.91 ± 1.30	<0.001	0.000
Neuron Count	4.20 ± 2.10	8.09 ± 1.76	11.36 ± 2.11	<0.001	0.001

Notes: Results were expressed as the number of positive cells per high-power field (cells/HPF).

## Data Availability

The original contributions presented in this study are included in the article. Further inquiries can be directed to the corresponding author.

## Abbreviations

The following abbreviations are used in this manuscript:

ARRIVE	Animal Research: Reporting of In Vivo Experiments
BBB	Blood–Brain Barrier
H&E	Hematoxylin and Eosin
ICH	Intracerebral Hemorrhage
IL-1β	Interleukin-1 beta
MDA	Malondialdehyde
MMP-9	Matrix Metalloproteinase-9
NLRP3	NOD-, LRR-, and pyrin domain-containing protein 3
NRF2	Nuclear factor erythroid 2-related factor 2
ROS	Reactive Oxygen Species
SOD	Superoxide Dismutase
SOD1	Superoxide Dismutase 1
TNF-α	Tumor Necrosis Factor Alpha
TQ	Thymoquinone
